# Supplementary material for: The effects of genital myiasis on the diversity of the vaginal microbiota in female Bactrian camels
Source: BMC Vet Res. 2022 Mar 5;18:87. doi: 10.1186/s12917-022-03189-5 (PMC8897907; doi:10.1186/s12917-022-03189-5)
Supplement: Supplementary file 5 — Additional file 5. [file 12917_2022_3189_MOESM5_ESM.zip › MPL201709200_16s_yy/Treat1/css/js_css/jsPDF-master.zip!/jsPDF-master/docs/libs_html2pdf.js.html]

libs/html2pdf.js - Documentation


## Home

### Classes

- FontObject
- jsPDF
- PubSub

### Global

- addFont
- addMetadata
- addPage
- CapJoinStyles
- circle
- CssColors
- ellipse
- getFontList
- html2pdf
- http
- lines
- lstext
- output
- rect
- requirejs
- reset
- roundedRect
- save
- setDisplayMode
- setDrawColor
- setFillColor
- setFont
- setFontSize
- setFontStyle
- setLineCap
- setLineJoin
- setLineWidth
- setPage
- setProperties
- setTextColor
- text
- toUrl
- triangle
- triggerEvent

# libs/html2pdf.js

```
/**
 * html2pdf.js
 * Copyright (c) 2014 Steven Spungin (TwelveTone LLC)  steven@twelvetone.tv
 *
 * Licensed under the MIT License.
 * http://opensource.org/licenses/mit-license
 */

function html2pdf (html,pdf,callback) {
	var canvas = pdf.canvas;
	if (!canvas) {
		alert('jsPDF canvas plugin not installed');
		return;
	}
	canvas.pdf = pdf;
	pdf.annotations = {

		_nameMap : [],

		createAnnotation : function(href,bounds) {
			var x = pdf.context2d._wrapX(bounds.left);
			var y = pdf.context2d._wrapY(bounds.top);
			var page = pdf.context2d._page(bounds.top);
			var options;
			var index = href.indexOf('#');
			if (index >= 0) {
				options = {
					name : href.substring(index + 1)
				};
			} else {
				options = {
					url : href
				};
			}
			pdf.link(x, y, bounds.right - bounds.left, bounds.bottom - bounds.top, options);
		},

		setName : function(name,bounds) {
			var x = pdf.context2d._wrapX(bounds.left);
			var y = pdf.context2d._wrapY(bounds.top);
			var page = pdf.context2d._page(bounds.top);
			this._nameMap[name] = {
				page : page,
				x : x,
				y : y
			};
		}

	};
	canvas.annotations = pdf.annotations;
	
	pdf.context2d._pageBreakAt = function(y) {
		this.pageBreaks.push(y);
	};

	pdf.context2d._gotoPage = function(pageOneBased) {
		while (pdf.internal.getNumberOfPages() < pageOneBased) {
			pdf.addPage();
		}
		pdf.setPage(pageOneBased);
	}

	if (typeof html === 'string') {
		// remove all scripts
		html = html.replace(/<script\b[^<]*(?:(?!<\/script>)<[^<]*)*<\/script>/gi, '');

		var iframe = document.createElement('iframe');
		//iframe.style.width = canvas.width;
		//iframe.src = "";
		//iframe.document.domain =
		document.body.appendChild(iframe);
		var doc;
		doc = iframe.contentDocument;
		if (doc == undefined || doc == null) {
			doc = iframe.contentWindow.document;
		}
		//iframe.setAttribute('style', 'position:absolute;right:0; top:0; bottom:0; height:100%; width:500px');

		doc.open();
		doc.write(html);
		doc.close();

		var promise = html2canvas(doc.body, {
			canvas : canvas,
			onrendered : function(canvas) {
				if (callback) {
					if (iframe) {
						iframe.parentElement.removeChild(iframe);
					}
					callback(pdf);
				}
			}
		});

	} else {
		var body = html;
		var promise = html2canvas(body, {
			canvas : canvas,
			onrendered : function(canvas) {
				if (callback) {
					if (iframe) {
						iframe.parentElement.removeChild(iframe);
					}
					callback(pdf);
				}
			}
		});
	}

}
```

  

Documentation generated by JSDoc 3.4.2 on Sat Oct 08 2016 21:42:26 GMT+0100 (BST) using the docdash theme.
